# Image Data Resource: a bioimage data integration and publication platform

**DOI:** 10.1038/nmeth.4326

**Published:** 2017-06-19

**Authors:** Eleanor Williams, Josh Moore, Simon W Li, Gabriella Rustici, Aleksandra Tarkowska, Anatole Chessel, Simone Leo, Bálint Antal, Richard K Ferguson, Ugis Sarkans, Alvis Brazma, Rafael E Carazo Salas, Jason R Swedlow

**Affiliations:** 1 Centre for Gene Regulation and Expression, University of Dundee, Dundee, UK; 2 Division of Computational Biology, University of Dundee, Dundee, UK; 3 European Molecular Biology Laboratory, European Bioinformatics Institute, Hinxton, UK; 4 Pharmacology Department, University of Cambridge, Cambridge, UK; 5 Genetics Department, University of Cambridge, Cambridge, UK; 6 Cambridge Systems Biology Centre, University of Cambridge, Cambridge, UK; 7 LOB, Ecole Polytechnique, CNRS, INSERM, Université Paris-Saclay, Palaiseau, France; 8 Center for Advanced Studies, Research, and Development in Sardinia (CRS4), Pula, Italy; 9 School of Cell and Molecular Medicine, University of Bristol, Bristol, UK

**Keywords:** Protein databases, Data integration, Protein databases

## Abstract

Access to primary research data is vital for the advancement of science. To extend the data types supported by community repositories, we built a prototype Image Data Resource (IDR). IDR links data from several imaging modalities, including high-content screening, multi-dimensional microscopy and digital pathology, with public genetic or chemical databases and cell and tissue phenotypes expressed using controlled ontologies. Using this integration, IDR facilitates the analysis of gene networks and reveals functional interactions that are inaccessible to individual studies. To enable reanalysis, we also established a computational resource based on Jupyter notebooks that allows remote access to the entire IDR. IDR is also an open-source platform for publishing imaging data. Thus IDR provides an online resource and a software infrastructure that promotes and extends publication and reanalysis of scientific image data.

**Supplementary information:**

The online version of this article (doi:10.1038/nmeth.4326) contains supplementary material, which is available to authorized users.

## Abstract (web summary)

This Resource describes the Image Data Resource (IDR), a prototype online system for biological image data that links experimental and analytic data across multiple data sets and promotes image data sharing and reanalysis.

**Supplementary information:**

The online version of this article (doi:10.1038/nmeth.4326) contains supplementary material, which is available to authorized users.

## Main

Much of the published research in the life sciences is based on image data sets that sample 3D space, time and the spectral characteristics of detected signal to provide quantitative measures of cell, tissue and organismal processes and structures. The sheer size of biological image data sets makes data submission, handling and publication challenging. An image-based genome-wide 'high-content' screen (HCS) may contain more than 1 million images, and new 'virtual slide' and 'light sheet' tissue imaging technologies generate individual images that contain gigapixels of data showing tissues or whole organisms at subcellular resolutions. At the same time, published versions of image data are often mere illustrations: they are presented in processed, compressed formats that cannot convey the measurements and multiple dimensions contained in the original image data and cannot easily be reanalyzed. Furthermore, conventional publications do not include the metadata that define imaging protocols, biological systems and perturbations or the processing and analytic outputs that convert the image data into quantitative measurements.

Several public image databases have appeared over the past few years. These provide online access to image data, enable browsing and visualization and, in some cases, include experimental metadata. The Allen Brain Atlas, the Human Protein Atlas and the Edinburgh Mouse Atlas all synthesize measurements of gene expression, protein localization and/or other analytic metadata with coordinate systems that place biomolecular localization and concentration into a spatial and biological context^1,2,3^. There are many other examples of dedicated databases for specific imaging projects, each tailored for specific aims and target communities^4,5,6,7,8^. A number of public resources serve as scientific, structured repositories for image data—i.e., they collect, store and provide persistent identifiers for long-term access to submitted data sets and provide rich functionalities for browsing, search and query. One archetype is the EMDataBank, the definitive community repository for molecular reconstructions recorded by electron microscopy^9^. The *Journal of Cell Biology* has built the JCB DataViewer, which publishes image data sets associated with its online publications. The CELL Image Library includes several thousand community-submitted images, some of which are linked to publications^10^. Figshare stores 2D pictures derived from image data sets and can provide links to download image data. The EMDataBank recently released a prototype repository for 3D tomograms, the EMPIAR resource^11^. Finally, the BioStudies and Dryad archives include support for browsing and downloading image data files linked to studies or publications^12^. Some of these provide a resource for a specific imaging domain (for example, EMDataBank) or experiment (MitoCheck), whereas others archive data sets and provide links to related publications at external journal websites (BioStudies). However, no existing resource links independent biological imaging data sets to provide an 'added-value' platform similar to Expression Atlas, for gene expression data^13^, or UniProt, for protein sequence and function data^14^.

Inspired by these added-value resources, we built IDR, an added-value platform that combines data from multiple independent imaging experiments and imaging modalities and integrates them into a single resource for reanalysis in a convenient, scalable form. IDR provides a prototyped resource that supports browsing, search, visualization and computational processing within and across data sets acquired from a wide variety of imaging domains. For each study, image data are stored along with metadata related to the experimental design, data acquisition and analysis and made available for search and query through a web interface and a single application program interface (API). Where possible, we have mapped the phenotypes determined by data set authors to a common ontology. For several studies, we have calculated comprehensive sets of image features that can be used by others for reanalysis and the development of phenotypic classifiers. By harmonizing data from multiple imaging studies into a single system, IDR enables users to query across studies and identify phenotypic links between different experiments and perturbations.

## Results

### Current IDR

IDR is currently populated with 24 imaging studies, comprising 35 screens or biological imaging experiments, most of which are linked to published works (Table 1). IDR holds ∼42 TB of image data in ∼36 million image planes and ∼1 million individual experiments and includes all associated experimental annotations (such as genes, RNAi, chemistry, geographic location), analytic annotations (submitter-calculated image regions and features) and functional annotations. Data sets from studies in human, mouse, fly, plant and fungal cells are included. The imaging modalities and experimental approaches supported include super-resolution 3DSIM and dSTORM, high-content chemical and siRNA screening, whole-slide histopathology imaging and live imaging of human and fungal cells and intact mice. Imaging data from Tara Oceans, a global survey of plankton and other marine organisms, are also included. The current collection samples biomedically relevant features such as cell shape, division and adhesion, from nanometer-scale localization of cellular proteins to millimeter-scale structures of animal tissues (Table 2).

**Table 1:** Data sets in IDR

Study identifier	Species	Type	Screens or experiments	5D images	Size (TB)	Pheno- types^a^	Targets^b^	Experiments^c^	Reference
idr0001-graml-sysgro	*S. pombe*	Gene deletion screen	1	109,728	10.06	19	3,005	18,432	^5^
idr0002-heriche-condensation	Human	RNAi screen	1	1,152	2.10	2	102	1,152	^26^
idr0003-breker-plasticity	*Saccharomyces cerevisiae*	Protein screen	1	97,920	0.20	14	6,234	32,640	^41^
idr0004-thorpe-rad52	*S. cerevisiae*	Gene deletion screen	1	3,765	0.17	1	4,195	4,512	^42^
idr0005-toret-adhesion	*Drosophila melanogaster*	RNAi screen	2	45,792	0.14	1	13,035	15,264	^43^
idr0006-fong-nuclearbodies	Human	Protein localization screen	1	240,848	1.40	8	12,743	16,224	^44^
idr0007-srikumar-sumo	*S. cerevisiae*	Protein localization screen	1	3,456	0.02	23	377	1,152	^45^
idr0008-rohn-actinome	*D. melanogaster*, human	RNAi screen	2	55,944	0.12	46	12,826	26,496	^40^
idr0009-simpson-secretion	Human	RNAi screen	2	397,056	3.25	3	17,960	397,056	^27^
idr0010-doil-dnadamage	Human	RNAi screen	1	56,832	0.08	2	18,675	56,832	^46^
idr0011-ledesmafernandez-dad4	*S. cerevisiae*	Gene deletion screen	5	8,957	0.4	1	5,209	8,736	NA
idr0012-fuchs-cellmorph	Human	RNAi screen	1	45,692	0.38	18	16,701	26,112	^39^
idr0013-neumann-mitocheck	Human	RNAi screen	2	200,995	14.54	18	18,393	206,592	^4^
idr0015-UNKNOWN-taraoceans	Multi-species	Geographic screen	1	32,776	2.49	0	84	84	^47^
idr0016-wawer- bioactivecompoundprofiling	Human	Small molecule screen	1	869,820	3.19	2	29,542	144,000	^48^
idr0017-breinig-drugscreen	Human	Small molecule screen	1	147,456	2.48	0	1,281	36,864	^49^
idr0018-neff-histopathology	*Mus musculus*	Histopathology of gene knockouts	1	899	0.27	48	9	248	—
idr0019-sero-nfkappab	Human	HCS image analysis	1	25,872	0.03	0	198	2,156	^50^
idr0020-barr-chtog	Human	RNAi screen	1	36,960	0.03	2	241	1,232	^51^
idr0021-lawo- pericentriolarmaterial	Human	Protein localization using 3D-SIM	1	414	0.0003	1	9	414	^52^
idr0023-szymborska- nuclearpore	Human	Protein localization using dSTORM	1	524	0.0005	1	7	359	^53^
idr0027-dickerson- chromatin	*S. cerevisiae*	3D-tracking of tagged chromatin loci	1	229	0.03	0	8	112	^54^
idr0028-pascualvargas-rhogtpases	Human	RNAi screen	4	155,332	0.18	9	170	5,544	^55^
idr0032-yang-meristem	*Arabidopsis thaliana*	*In situ* hybridization	1	458	0.003	5	115	115	^56^
Sum			35	2,538,777	42	224	161,119	1,002,328	
Average				105,782	1.73	9	6,713	41,764	

^a^The number of submitted phenotypes.

^b^The number of genes, compounds or proteins identified as targets for analysis.

^c^The number of individual wells (in HCS studies) or imaging experiments (in nonscreen data sets). NA, not applicable (unpublished data).

**Table 2:** Example URLs and views of IDR data sets

Study identifier	IDR URL
idr0001-graml-sysgro	https://idr.openmicroscopy.org/webclient/?show=well-590686
idr0002-heriche-condensation	https://idr.openmicroscopy.org/webclient/?show=well-119093
idr0003-breker-plasticity	https://idr.openmicroscopy.org/webclient/?show=well-4852
idr0004-thorpe-rad52	https://idr.openmicroscopy.org/webclient/?show=well-469267
idr0005-toret-adhesion	https://idr.openmicroscopy.org/webclient/?show=well-547609
idr0006-fong-nuclearbodies	https://idr.openmicroscopy.org/webclient/?show=image-820684
idr0007-srikumar-sumo	https://idr.openmicroscopy.org/webclient/?show=well-37472
idr0008-rohn-actinome	https://idr.openmicroscopy.org/webclient/?show=well-45407
idr0009-simpson-secretion	https://idr.openmicroscopy.org/webclient/?show=image-648950
idr0010-doil-dnadamage	https://idr.openmicroscopy.org/webclient/?show=image-3063667
idr0011-ledesmafernandez-dad4	https://idr.openmicroscopy.org/webclient/?show=image-2849866
idr0012-fuchs-cellmorph	https://idr.openmicroscopy.org/webclient/?show=image-1821818
idr0013-neumann-mitocheck	https://idr.openmicroscopy.org/webclient/?show=image-1636543
idr0015-UNKNOWN-taraoceans	https://idr.openmicroscopy.org/webclient/?show=well-1056578
idr0016-wawer-bioactivecompoundprofiling	https://idr.openmicroscopy.org/webclient/?show=well-1029401
idr0017-breinig-drugscreen	https://idr.openmicroscopy.org/webclient/?show=well-1046336
idr0018-neff-histopathology	https://idr.openmicroscopy.org/webclient/?show=dataset-369
idr0019-sero-nfkappab	https://idr.openmicroscopy.org/webclient/?show=well-1024671
idr0020-barr-chtog	https://idr.openmicroscopy.org/webclient/?show=well-1030579
idr0021-lawo-pericentriolarmaterial	https://idr.openmicroscopy.org/webclient/?show=dataset-51
idr0023-szymborska-nuclearpore	https://idr.openmicroscopy.org/webclient/?show=dataset-61
idr0027-dickerson-chromatin	https://idr.openmicroscopy.org/webclient/?show=image-2858266
idr0028-pascualvargas-rhogtpases	https://idr.openmicroscopy.org/webclient/?show=image-2895051
idr0032-yang-meristem	https://idr.openmicroscopy.org/webclient/?show=image-3125776

### Genetic, chemical and functional annotation in IDR

To enable querying across data sets in IDR, we have included annotations describing experimental perturbations (such as genetic mutants, siRNA targets and reagents, expressed proteins, cell lines and drugs) and phenotypes declared by study authors either from quantitative analysis or visual inspection of image data. Where possible, experimental metadata in IDR link to authoritative external resources (such as Ensembl, NCBI or PubChem).

Many of the studies in IDR perturb gene function by mutation or siRNA depletion. To calculate the sampling of gene orthologs, we used Ensembl's BioMart resource^15^ to access a normalized list of gene orthologs. Overall, 19,601 gene orthologs are sampled; of these, 84.1% are sampled more than 20 times and 90.3% are sampled in three or more studies. Even in this early incarnation, the phenotypes of perturbations in the majority of known genes are sampled in several assays and organisms.

**Figure 1:**
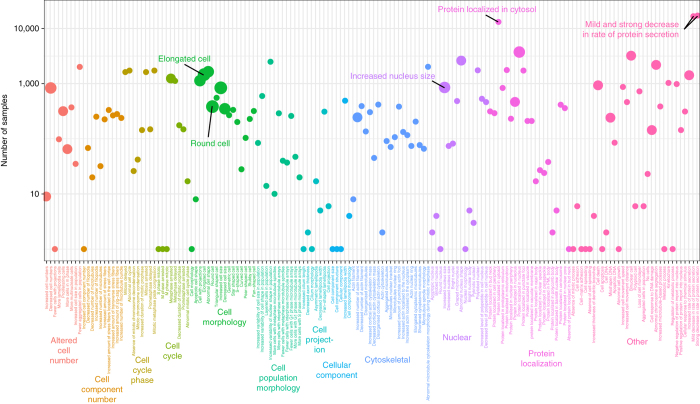
Sampling of phenotypes in the IDR. Each sample represents a well from a microwell plate in a screen or an image from a data set. Wells annotated as controls were not included. User-submitted phenotype terms were mapped to the CMPO terms shown here. Colors represent higher-level groupings of phenotype terms. Point size represents the number of studies each phenotype is linked to (1, 2, 3 or 4 studies).

We normalized the phenotypes included in studies submitted to IDR. Functional annotations were converted to defined terms in the Cellular Microscopy Phenotype Ontology (CMPO)^16^ or other ontologies, in collaboration with the data submitters. Overall, 88% of the functional annotations have links to defined, published controlled vocabularies. IDR includes 158 ontology-normalized phenotypes (for example, 'increased number of actin filaments' and 'mitosis arrested'), and 136 are reported in only one study. Nonetheless, these phenotypes were well sampled, with an average of 698 samples per phenotype across HCSs and other imaging data sets, and a median of 144. This skewing occurs because some phenotypes are very common or over-represented in specific assays, for example, 'protein localized in cytosol phenotype' (CMPO_0000393). Nonetheless, several phenotypes were observed in multiple orthogonal assays (e.g., 'round cell' (CMPO_0000118) and 'increased nuclear size' (CMPO_0000140)). Figure 1 summarizes the sampling of phenotypes across the current IDR data sets. Several classes of phenotypes are included, and many cases are sampled in thousands of experiments. In total, IDR includes >1 million individual experiments (Table 1), ∼9% of which are annotated with experimentally observed phenotypes.

### Data visualization in IDR

IDR integrates image data and metadata from several studies. The current IDR web user interface (WUI) is based on OMERO.web, an open-source application^17^, and is supplemented with a plugin allowing data sets to be viewed by study, genes, phenotypes, siRNAs, antibodies, compounds and organisms (Supplementary Note). This architecture makes the integrated data resource available for access and reuse in several ways (Supplementary Note). Image data are viewable as thumbnails for each study, and multidimensional images can be viewed and browsed. Tiled whole-slide images used in histopathology are also supported. Any regions of interest (ROIs) submitted with the image data are included and linked and, where possible, made available through the IDR WUI. IDR images, thumbnails and metadata are accessible through the IDR WUI and web-based API in JSON format (Supplementary Note). They also can be embedded into other pages (e.g., Euro-BioImaging, (https://www.eurobioimaging-interim.eu/image-data-resource.html) using the OMERO.web gateway.

### Standardized interfaces for imaging metadata

IDR integrates imaging data from many studies. These data were acquired by various imaging modalities, in the absence of overarching standards for experimental, imaging or analytic metadata. While efforts such as MIACA (http://miaca.sourceforge.net/), NeuroVault^18^, MULTIMOT^19^ have proposed data standards in specific imaging subdomains, there is not yet a metadata standard that crosses all the imaging domains potentially served by IDR. We therefore sought to adopt lightweight methods from other communities that have had broad acceptance^20^ and converted metadata submitted in custom formats—spreadsheets, PDFs, MySQL databases and Microsoft Word documents—into a consistent tabular format, inspired by the MAGE-TAB and ISA-TAB specifications^21,22^, that could then be used for importing semistructured metadata such as gene and ontology identifiers into OMERO^23^. We also used the Bio-Formats software library to identify and convert well-defined, semantically typed elements that describe imaging metadata (for example, image pixel size) as specified in the OME Data Model^24,25^, and we used the resulting translation scripts to integrate data sets into a single resource. The scripts are publicly available (Online Methods) and thus comprise a framework for recognizing and reading a range of metadata types across several imaging domains into a common, open specification.

### Added value of IDR

Because IDR links gene names and phenotypes, query results that combine genes and phenotypes across multiple studies are possible through simple text-based search. Searching for the gene *SGOL1* (https://idr.openmicroscopy.org/mapr/gene/?value=SGOL1) returns a range of phenotypes from four studies associated with mitotic defects (for example, CMPO_0000118, CMPO_0000305, CMPO_0000212 and CMPO_0000344)^4,26^ but also an accelerated secretion phenotype (CMPO_0000246) in a screen for defects in protein secretion^27^. A second example is provided in a histopathology study of tissue phenotypes in a series of mouse mutants. Knockout of *Car4*, which encodes carbonic anhydrase 4 in mouse, results in a range of defects in homeostasis in the brain, rib growth and male fertility^28,29,30^. Data in IDR show abnormal nuclear phenotypes in several tissues from *Car4*^−/−^ mice, including gastrointestinal (https://idr.openmicroscopy.org/webclient/?show=dataset-153), liver (https://idr.openmicroscopy.org/webclient/?show=image-1918940) and male reproductive tract (https://idr.openmicroscopy.org/webclient/?show=image-1918953). The human ortholog, *CA4*, is involved in certain forms of retinitis pigmentosa^31,32^. Data in IDR from the MitoCheck study show that siRNA-mediated depletion of CA4 in HeLa cells^4^ also results in abnormally shaped nuclei (https://idr.openmicroscopy.org/webclient/?show=well-828419), consistent with a defect in some aspect of the cell division cycle.

**Figure 2:**
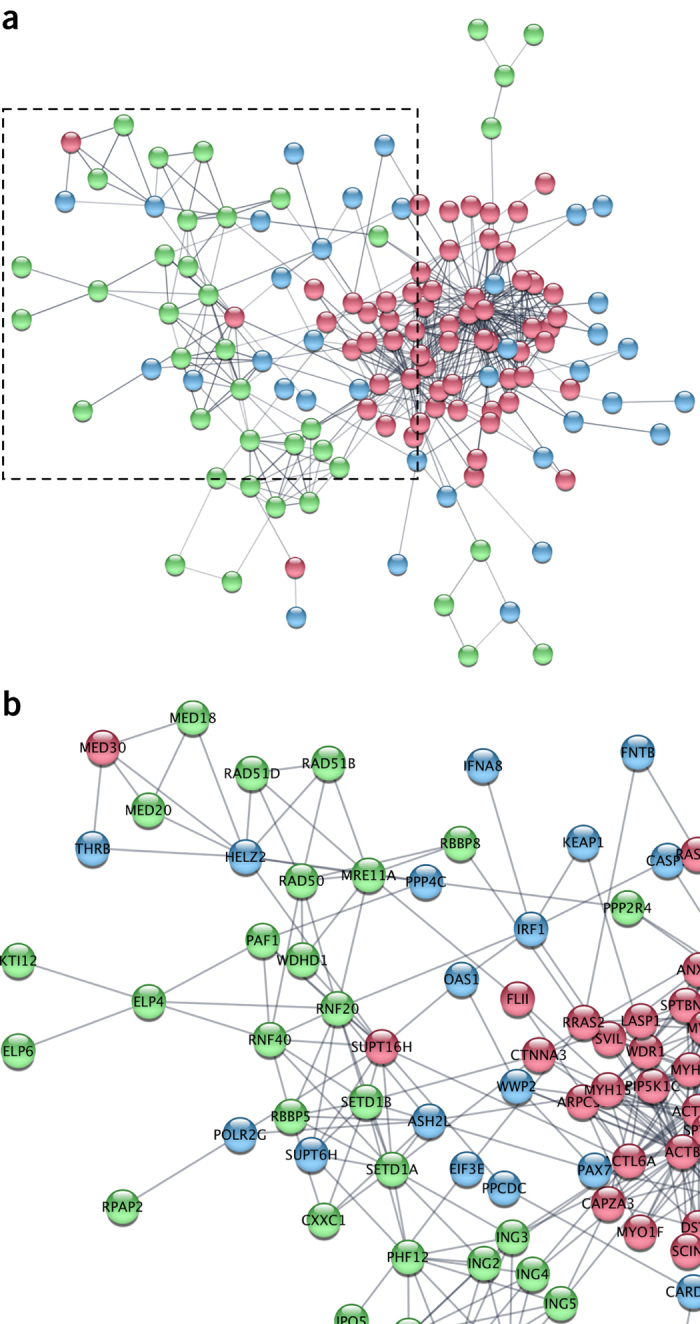
Network analysis of genes linked to the elongated cell phenotype in the IDR. (**a**) Protein–protein interaction network based on the genes linked to the elongated cell phenotype (CMPO_0000077) in three IDR studies. Genes from *S. pombe* (green, idr0001-A)^5^, HeLa cell morphology (blue, idr0012-A)^39^ and HeLa Actinome (red, idr0008-B)^40^ are displayed with linkages (gray) from StringDB^33^. To enable comparisons in Cytoscape, the human orthologs of *S. pombe* genes are used for the genes identified in idr0001-A (Supplementary Note). (**b**) Close-up view of network in **a**. Genes are listed in Supplementary Note.

Phenotypes across distinct studies can also be used to build novel representations of gene networks. Figure 2a shows the gene network created when knockouts or knockdowns that caused an elongated cell phenotype (CMPO_0000077) in *Schizosaccharomyces pombe* and human cells are linked by queries to String DB^33^ and visualized in Cytoscape^34^ (Supplementary Note and Supplementary Table 1). The genes discovered in the three studies form nonoverlapping, complementary networks that connect specific macromolecular complexes to the elongated cell phenotype. For example, HELZ2, MED30, MED18 and MED20 are all part of the mediator complex but were identified as 'elongated cell' hits in separate studies using different biological models (idr0001-A, idr0008-B and idr0012-A) (Fig. 2b). POLR2G (idr0012-A), PAF1 (idr0001-A) and SUPT16H (idr0008-B) were scored as elongated cell hits in these studies and are all part of the elongation complex in the RNA polymerase II transcription pathway. Finally, ASH2L (elongated cell phenotype in idr0012-A), associates with SETD1A and SETD1B (elongated cell phenotype in idr0001-A) to form the Set1 histone methyltransferase (HMT). These examples show that the individual hits are probably not due to off-target effects or characteristics of individual biological models but arise through conserved, specific functions of large macromolecular complexes.

The integration of experimental, imaging and analytic metadata also provides an opportunity to include new functionalities for data visualization and analysis, adding further value to the original studies and data sets. We have added the data analytics tool Mineotaur^35^ to one of IDR's data sets (https://idr.openmicroscopy.org/mineotaur/). This allows visual querying and analysis of quantitative feature data. For instance, having shown that components of the Set1 HMT function in controlling cell morphology in *S. pombe* and human cells, we noticed that genes such as *ASH2L* were in the 'elongated cell' network based on human cell data (idr0012-A) but not *S. pombe* data. We noted that *ash2* has a microtubule cytoskeleton phenotype (https://idr.openmicroscopy.org/webclient/?show=well-592371), then we queried the criteria used for cell shape hits in the Sysgro screen (idr0001-A) and found that *ash2* fell just below the cutoff originally used in this study to define phenotypic hits for cell shape (Supplementary Note). When combined with results on *ASH2L* from HeLa cells (Fig. 2b) these results suggest that the Set1 HMT has a strongly conserved role in controlling cell shape and the cytoskeleton in unicellular and multicellular organisms.

### Data integration and access

Like most modern online resources, IDR makes data available through a web user interface as well as a web-based JSON API. This encourages third parties to make use of IDR on their own sites. For example, image data in IDR have been linked to study data in BioStudies (for example, BioStudies S-EPMC4704494) and to PhenoImageShare^36^, an online phenotypic repository (http://www.phenoimageshare.org/search/?term=&hostName=Image+Data+Repository+(IDR)).

To further extend the possibilities for reuse of IDR data, we are calculating comprehensive sets of feature vectors of IDR image data using the open-source tool WND-CHARM^37^. To date, full WND-CHARM features have been calculated for images in idr0002-A, idr0005-A, idr0008-B, idr0009-A, idr0009-B and idr0012-A and for parts of idr0013-A and idr0013-B. Features are stored in IDR using OMERO's HDF5-based data store and available through the OMERO API (Supplementary Note).

The integration of image-based phenotypes and calculated features makes IDR an attractive candidate for computational reanalysis. To ease the access to IDR's TB-scale data sets, we have connected IDR to a Jupyter notebook-based computational resource (https://idr.openmicroscopy.org/jupyter) that exposes IDR data sets via an API (https://idr.openmicroscopy.org/about/api.html). We include example notebooks that provide visualization of image features using PCA, access to images annotated with CMPO phenotypes, calculations of gene networks and WND-CHARM features for individual images and recreation of Figures 1 and 2 from IDR data. Users can also run their own analyses using notebooks stored in GitHub (https://github.com/IDR/idr-notebooks). To allow reuse of IDR metadata locally, we have made all IDR databases, metadata and thumbnails available for download and have built Ansible scripts that automate deployment of the IDR software stack (original image data are not included; see Supplementary Note).

## Discussion

Making data public and available is a critical part of the scientific enterprise^38^. To help facilitate the reuse and meta-analysis of image data sets, we have built IDR, a next-generation data technology that integrates and publishes image data and metadata from a wide range of imaging modalities and scales in a consistent format. IDR integrates experimental, imaging, phenotypic and analytic metadata from several independent studies into a single resource, allowing new modes of biological Big Data querying and analysis. As more data sets are added to IDR, they will potentiate and catalyze the generation of new biological hypotheses and discoveries.

In IDR, we have linked image metadata from several independent studies. Experimental, imaging phenotypic and analytic metadata are recorded in a consistent format. Rather than attempting to enforce a strict imaging data standard, IDR provides tools for supporting community formats and releases these as a framework that facilitates data reuse. We hope that the availability of this framework will provide incentives for others to structure metadata in shareable formats that can be read into IDR or other applications. In the future, we can imagine that these and other capabilities could be extended in IDR—or similar repositories that link to IDR—to enable systematic integration, visualization and analytics across imaging studies, thereby helping to harness and capitalize on the increasing amounts of bioimaging data that the community generates.

As of this writing, IDR has published 35 reference image data sets grouped into 24 studies (Table 1) and, using EMBL-EBI's Embassy Cloud, has the capacity to receive and publish many more. Authors can submit image data sets for publication in IDR using the metadata specifications and formats we have built (details about the submission process are available at https://idr.openmicroscopy.org/about/submission.html). Once published, the data sets can be browsed and viewed through IDR's WUI or queried and reanalyzed using the IDR computational resource.

IDR software and technology is open source, so it can be accessed and built into other systems for image data publication. This supports the building of technology and installations that integrate and publish bioimaging data for the scientific community. IDR therefore functions both as a resource for image data publication and as a technology platform that supports online scientific image databases and services. In the future, those databases and services may amalgamate to form resources analogous to the genomic resources that are the foundation of much of modern biology.

## Methods

### Architecture and population of IDR.

IDR (https://idr.openmicroscopy.org) was built using open-source OMERO^17^ and Bio-Formats^24^ as a foundation. Deployments are managed by Ansible playbooks along with re-usable roles on an OpenStack-based cloud contained within the EMBL-EBI Embassy resource. Data sets (Table 1) were collected by shipped USB drive or transferred by Aspera. Included data sets were selected according to the criteria defined by the Euro-BioImaging/Elixir Data Strategy concept of reference images (http://www.eurobioimaging.eu/content-news/euro-bioimaging-elixir-image-data-strategy), which states that image data sets for publication should be related to published studies, linked as much as possible to other resources and candidates for reuse, reanalysis and/or integration with other studies.

Experimental and analytic metadata were submitted in spreadsheets (CSV, XLS), PDF or HDF5 format or a MySQL database, each using its own custom format. We converted these custom formats to a consistent tabular format inspired by the MAGE and ISA-TAB specifications^21,22^ and combined them into a single CSV file using a custom script and imported into OMERO. Imaging metadata and binary data were imported into OMERO using Bio-Formats. Experimental and analytic metadata were stored using OMERO.tables, an HDF5-backed tabular data store used by OMERO. For each data set, metadata that were valuable for querying and search were copied to OMERO's key-value-based Map Annotation facility^23^. This means that different metadata types and elements can be accessed using different parts of the OMERO API, depending on the search and querying capabilities they require. For more information on the construction of queries, see Supplementary Note.

### Code availability.

All software for building and running the IDR and reading metadata of the IDR data sets is open source and available at https://github.com/IDR and https://github.com/openmicroscopy. The custom scripts used to combine metadata into a single CSV files are available at https://github.com/IDR/idr-metadata.

### Data availability.

All data sets described in this paper are available at https://idr.openmicroscopy.org.

## Additional information

**Publisher's note:** Springer Nature remains neutral with regard to jurisdictional claims in published maps and institutional affiliations.

## Supplementary information

Supplementary Text and Figures Supplementary Note. (PDF 1279 kb)

Supplementary Table 1 Genes linked to the elongated cell phenotype in IDR. (XLSX 21 kb)
