# Supplementary material for: Image Data Resource: a bioimage data integration and publication platform
Source: Nat Methods. 2017 Jun 19;14(8):775–81. doi: 10.1038/nmeth.4326 (PMC5536224; doi:10.1038/nmeth.4326)
Supplement: Supplementary Text and Figures — Supplementary Note. (PDF 1279 kb) [file 41592_2017_BFnmeth4326_MOESM1_ESM.pdf]

Supplementary Note-- Williams et al

The Image Data Resource: A Bioimage Data Integration and Publication Platform

1. Exploring the IDR

This current IDR web user interface (WUI) is based on the open source OMERO.web Django Python Web application[1] supplemented with a plugin called MAPR (https://github.com/ome/omero-mapr) that allows datasets to be viewed by Studies and custom attributes: Genes, Phenotypes, siRNAs, Compounds, and Organisms. Custom attributes are predefined as a set of primary keywords when experimental metadata (https://github.com/IDR/idr-metadata) are populated for each individual study and can be configured accordingly. Using this architecture makes the integrated data resource available for view, access and reuse in several ways.

1.1 Browsing the IDR

The default "Studies view" (http://idr.openmicroscopy.org/webclient/userdata/?experimenter=-1) lists all available datasets in the left-hand panel. When a study is selected in the tree, details on the study can be seen in the right hand panel, including hyperlinks to the original papers in the Attributes section. When data from the study is selected in the tree, the thumbnails are displayed in the center panel. Selecting a thumbnail displays the image details, all the metadata, and other information in the right-hand panel (e.g. click study idr0006-fong-nuclearbodies/screenA, click plate 11068, click well E04; URL to go to this well directly is http://idr.openmicroscopy.org/webclient/?show=well-451890). Experimental metadata associated with each study are loaded into the Attributes section. The MAPR plugin dynamically processes them according to the configuration, and this results in the conversion of declared primary keywords into interactive links. By clicking on a value, the user switches the context to filter the data based on the given value. In this way, the user can glance at images across multiple datasets annotated with the given attribute, seeking a desired concept or connections to other attributes that again can be used to switch the filtering context.

As an example, browse to well E04, plate 11068 in study idr0006-fong-nuclearbodies/screenA as described above. In the right panel, click on "Gene Symbol: ASH2L" (Screenshot 2). This changes the context in the left panel to search for Genes, and displays all studies that include the gene *ASH2L* (Screenshot 1; the same result is achieved with the URL: http://idr.openmicroscopy.org/webclient/?show=gene-ASH2L). Click study idr0012-fuchs-cellmorph/screenA, click plate HT28. Clicking either of the images reveals associated experimental, image and phenotypic metadata. Under Phenotypes, click on "CMPO_0000077" (Screenshot 3; the same results is achieved with the URL:

Nature Methods: doi:10.1038/nmeth.4326

http://idr.openmicroscopy.org/webclient/?show=phenotype-CMPO_0000077), and the WUI now reports all studies annotated with this CMPO term, “elongated cells phenotype”.

The current IDR web user interface (WUI) is based on the open source OMERO.web Django Python Web application[1] supplemented with a plugin called MAPR that allows datasets to be viewed by custom attributes: Study, Genes, Phenotypes, siRNAs, Compounds, and Organisms.

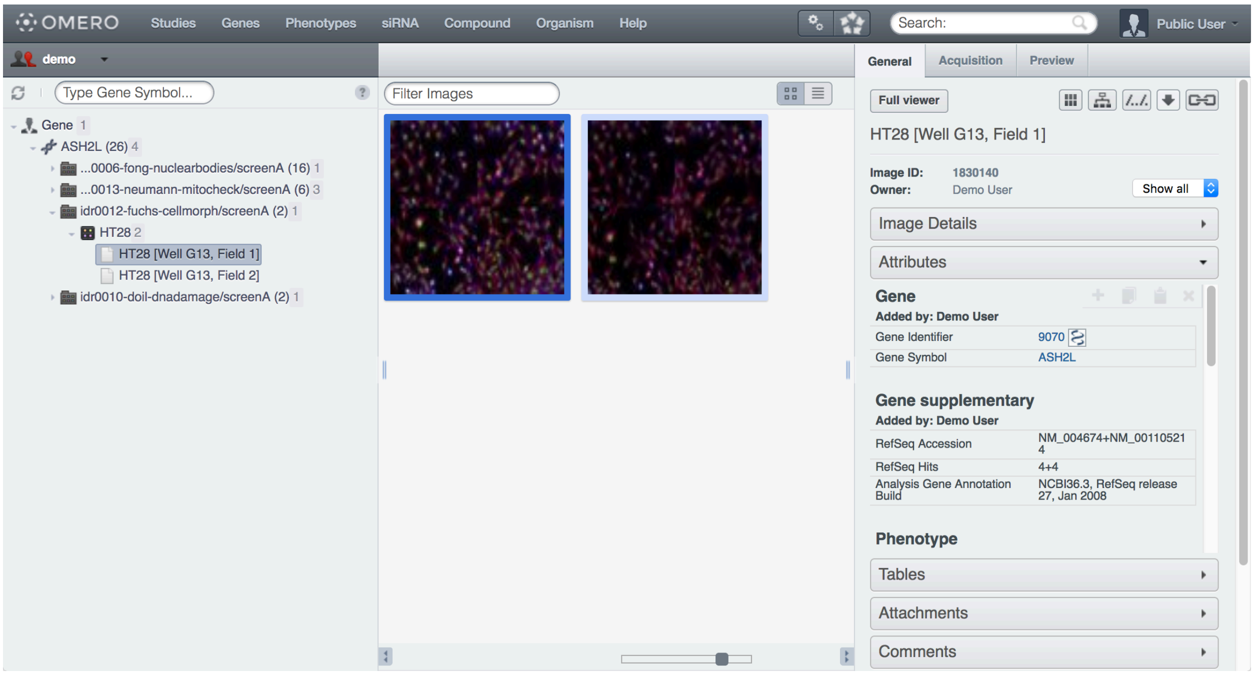

Screenshot 1. View of the IDR Interface after a query for the gene *ASH2L*. URL = http://idr.openmicroscopy.org/webclient/?show=gene-ASH2L .

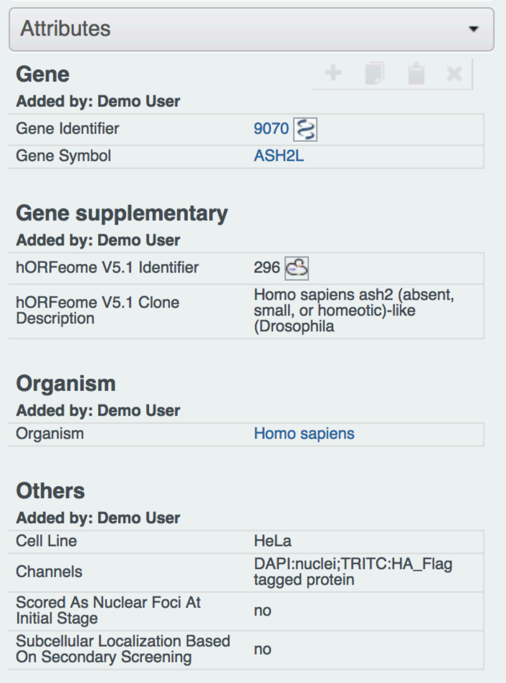

Screenshot 2. Metadata displayed for the *ASH2L* gene.

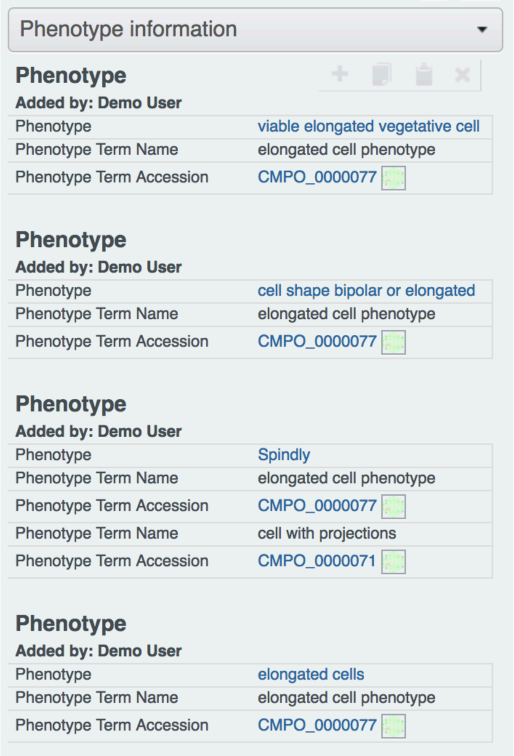

Screenshot 3. Metadata displayed after query for CMPO_0000077.

1.2 Linking IDR data

Each item in the data hierarchy benefits from a unique reference to an IDR web resource, which specifies its location in the repository and a mechanism for retrieving it. MAPR extends these references by additional links to the experimental metadata that were pre-configured in the plugin.

Example URLs:

1. Image link: http://idr.openmicroscopy.org/webclient/?show=image-122770
2. Gene link http://idr.openmicroscopy.org/webclient/?show=gene-ASH2L
3. Phenotype link http://idr.openmicroscopy.org/webclient/?show=phenotype-CMPO_0000077 or http://idr.openmicroscopy.org/webclient/?show=phenotype-elongated%20cells
4. Query http://idr.openmicroscopy.org/mapr/phenotype/?value=elongated&query=true

Table 1 summarises the use of these resources.

**URL**	**URL PARAMETERS**	**QUERY STRING**	**METHOD**	**SUCCESS RESPONSE**

<table>
<tr><td>/webclient/?show=<type>-<value></td><td></td><td>Required:
type=[string]</td><td>GET</td><td>302 Redirect</td></tr>
<tr><td>/mapr/<type>/?value=<value>
/mapr/<type>/?value=<value>&query=<query></td><td>Required:
type=[gene|phenotype|sirna|compound|organism]</td><td>Required:
value=[string]
Optional:
query=[boolean]
default:false</td><td>GET</td><td>200 HTML</td></tr>
</table>

Table 1. Query structure in IDR interface.

1.3 IDR Web API

IDR datasets, thumbnails and metadata are accessible through the IDR WUI and web-based API in JSON format. The full API specification for MAPR is available from https://github.com/ome/omero-mapr/tree/master/docs. Further API documentation for the IDR including an example script to load data from IDR is available on http://idr.openmicroscopy.org/about/api.html.

2. Embedding IDR images in external websites

IDR images and thumbnails can be embedded into other pages using the OMERO.web gateway (e.g., https://www.eurobioimaging-interim.eu/image-data-resource.html). Step by step example is available on http://www.openmicroscopy.org/site/support/omero5.3/developers/Web/ViewPort.html.

3. IDR Deployment

The current version of the IDR is hosted by EMBL-EBI on their Embassy OpenStack Cloud. The IDR is deployed using Ansible, an open-source configuration management system (https://www.ansible.com/). All Ansible playbooks and roles for building the IDR, with the exception of some private configuration details, are available in the GitHub “deployment” repository (https://github.com/idr/deployment). They have been tested with multiple OpenStack cloud platforms, though they should also work with most other cloud providers or physical infrastructure.

To set up a copy of the IDR infrastructure, first create your servers (virtual or physical) that will be used for hosting the IDR, and run the Ansible playbooks to deploy the IDR. If you are using OpenStack an example playbook is included to create the virtual machines and storage volumes. Playbooks are also included to setup the IDR analysis platform using Ansible Playbooks. Full documentation of the IDR set up is available at http://idr.openmicroscopy.org/about/deployment.html.

4. Downloading IDR Metadata and Thumbnails

If you wish to access a copy of the IDR databases, for example to populate a copy of the IDR infrastructure with IDR metadata, you must download the OMERO repository and the Postgres database. This will allow you to browse the image thumbnails, browse, search and query all IDR metadata in your copy of the IDR, however it does not include the original images files due to the size (around 40 TB). To download the IDR databases, use the following command in a terminal window:

```
rsync -rdt rsync://idr.openmicroscopy.org
```

and follow the information provided by the rsync command.

5. Adding your own data

The import of data and creation of metadata in the IDR is handled through definition files (https://github.com/IDR/idr-metadata). Each study is stored in its own directory, beginning with its IDR accession number. All information required for a study is held in this directory, ensuring the process of importing new data is completely reproducible. This metadata may include:

- Overview of the study, including authors, related publications, organisms, imaging modalities, etc.
- List of image files and organisational details
- Image, Well, Plate and Screen metadata that should be displayed in the IDR alongside when an object is viewed.
- Display settings for images (e.g. channel colours, intensity scaling)
- Features (obtained from the authors, or from published image analysis algorithms)
- Ontological terms such as genes and phenotypes, which can be browsed in the IDR.

6. IDR Analysis Platform

The IDR analysis platform is based around JupyterHub (http://jupyter.org/), which provides a cross-platform browser-based interface for analysing the IDR data in Python. This takes advantage of the orchestration support included in Docker (Docker Swarm ; https://www.docker.com/products/docker-swarm) to distribute user-environments across multiple servers. Authentication is currently managed by GitHub OAuth; users may be granted access to the platform following receipt of their GitHub username; send an email with the subject line REQUEST AUTHORIZATION to idr@openmicroscopy.org .

A set of IPython Notebooks are included to demonstrate how to access the IDR images and metadata, including features and all descriptive tags (https://github.com/IDR/idr-notebooks). Full access to IDR metadata and images is provided through the standard OMERO API (http://www.openmicroscopy.org/site/support/omero5/developers/). The notebooks in this repository are meant to exemplify the use of that API in the context of the IDR using the scipy stack Python library for data analysis (http://scipy.org); in particular they provide interactive versions of Fig. 1 and Fig. 2 of this paper using cytoscape.js (http://js.cytoscape.org) and bokeh (http://bokeh.pydata.org) respectively.

7. Genes Linked to the Elongated Cell Phenotype in IDR

Supplementary Table 1 shows genes linked to the elongated cell phenotype in IDR, listing the source screen, gene identifier used in the screen, equivalent Ensembl gene identifier and human orthologues of *S. pombe* genes from screen idr0001-A. Ensembl's BioMart resource[2] was used to obtain mappings between gene identifiers and human orthologues from StringDB[3]. Genes linked to the elongated cell phenotype, CMPO_000077, from three distinct studies stored in IDR were entered into StringDB and visualized using Cytoscape[4] in Figure 2. It is likely that in the future IDR will receive more submissions that include related phenotypic annotations, so Figure 2 shows one view of a network, based on an evolving set of data. IDR study idr0028 (http://idr.openmicroscopy.org/webclient/?show=screen-1502) also contains experiments annotated with CMPO_0000077/elongated cell phenotype. However this study used targeted libraries, so when included in the network, shows no novel functional interactions. Therefore, data from idr0028 have been omitted from this network, but can be viewed and queried interactively (http://idr.openmicroscopy.org/mapr/phenotype/?value=CMPO_0000077) or accessed using the IDR computational resource (https://idr.openmicroscopy.org/jupyter).

8. References

1. Allan, C. et al. OMERO: flexible, model-driven data management for experimental biology. *Nat Methods* **9**, 245-253 (2012).
2. Yates, A. et al. Ensembl 2016. *Nucleic Acids Res* **44**, D710-716 (2016).
3. Szklarczyk, D. et al. STRING v10: protein-protein interaction networks, integrated over the tree of life. *Nucleic Acids Res* **43**, D447-452 (2015).
4. Cline, M.S. et al. Integration of biological networks and gene expression data using Cytoscape. *Nat Protoc* **2**, 2366-2382 (2007).
